# Combinatorial control of *Spo11* alternative splicing by modulation of RNA polymerase II dynamics and splicing factor recruitment during meiosis

**DOI:** 10.1038/s41419-020-2443-y

**Published:** 2020-04-17

**Authors:** Eleonora Cesari, Maria Loiarro, Chiara Naro, Marco Pieraccioli, Donatella Farini, Livia Pellegrini, Vittoria Pagliarini, Pamela Bielli, Claudio Sette

**Affiliations:** 1 Department of Neuroscience, Section of Human Anatomy, Catholic University of the Sacred Heart, 00168 Rome, Italy; 2 Laboratory of Neuroembryology, IRCCS Fondazione Santa Lucia, 00143 Rome, Italy; 3 Department of Biomedicine and Prevention, University of Rome Tor Vergata, 00133 Rome, Italy

**Keywords:** Spermatogenesis, RNA splicing

## Abstract

Homologous recombination and chromosome segregation in meiosis rely on the timely expression of two splice variants of the endonuclease SPO11, named α and β, which respectively skip or include exon 2. However, in spite of its physiological importance, the mechanism underlying *Spo11* alternative splicing in meiosis is still unknown. By screening the activity of factors that are predicted to bind the alternatively spliced region of *Spo11*, we identified hnRNPH as a key regulator of SPO11α splicing in mouse spermatocytes. Although hnRNPH was not upregulated in meiosis concomitantly with the switch in splicing, its recruitment to *Spo11* pre-mRNA was favored by selective modulation of RNA polymerase II (RNAPII) phosphorylation and processivity in proximity of exon 2. The hnRNPH binding sites were localized near those of splicing factors that promote SPO11β splicing, suggesting that hnRNPH favors exon 2 skipping by competing out positive regulators. Indeed, hnRNPH binds proximal to a consensus motif for Sam68, a positive regulator of SPO11β splicing in vitro and in vivo, and it interferes with Sam68 binding to the *Spo11* pre-mRNA. Thus, our work reveals that modulation of RNAPII dynamics in concert with hnRNPH recruitment exerts a combinatorial control of the timely regulated *Spo11* splicing during meiosis.

## Introduction

Alternative splicing (AS) of pre-mRNAs is a powerful combinatorial mechanism that allows production of protein isoforms with different functions from each gene^1,2^. AS modularity allows expansion of the coding potential of the genome and promotes plasticity in its utilization^1^. Splicing reactions are orchestrated by a ribonucleoprotein complex called “spliceosome”, which recognizes exon–intron junctions, excises introns and ligates exons^3^. The lack of stringent consensus sequences at splice junctions in higher eukaryotes allows flexibility in their recognition. Numerous RNA-binding proteins (RBPs) act as splicing factors by interacting with the spliceosome, and reinforce or weaken recognition of exon–intron junctions. The interplay between these antagonistic splicing factors determines the choice of regulated exons by the spliceosome and causes heterogeneity in pre-mRNA processing^1,3,4^. In addition, AS is modulated by the rate of transcription and by epigenetic marks that decorate exons subject to regulation^5,6^. As a consequence, changes in the expression and/or in the activity of any of the factors contributing to these processes can selectively influence AS regulation. In particular, the elongation rate of the RNA polymerase II (RNAPII) is a key factor in co-transcriptional selection of regulated exons. A fast elongation rate makes more splice sites concomitantly available within a pre-mRNA, allowing competition between them and generally favoring stronger splice sites, whereas a slow rate of elongation promotes inclusion of weak exons^5,7^. However, genome-wide analyses indicated that several exons were also preferentially skipped by slowing down the RNAPII^8^. Such effect could rely on preferential recruitment of repressive splicing factors by the slow polymerase in the proximity of the regulated exon, as demonstrated for the *CFTR* gene^9^. Nevertheless, when and how such specific coordination is achieved under physiological situations remains largely unknown.

Testis is the organ displaying the highest abundance of splice variants^10^. In particular, meiotic spermatocytes display exceptional transcriptional and splicing diversity^10–12^. AS flexibility is exploited by meiotic cells to produce protein variants uniquely required for the peculiar processes involved in germ cell differentiation^13,14^, but also to dictate the timing of their expression^12^. An interesting example in this sense is offered by the *Spo11* gene, encoding the essential endonuclease that establishes DNA double strand breaks (DSBs) and initiates homologous recombination in meiosis^15,16^. The *Spo11* gene encodes for two main protein isoforms (SPO11α and β) which differ for exon 2 skipping (α) or inclusion (β). Early meiotic spermatocytes synthesize primarily SPO11β, whereas SPO11α becomes predominant in late meiosis^17^. Notably, the timing of *Spo11* AS parallels that of DSB formation during meiosis, with a first wave in leptotene/zygotene spermatocytes that affects autosomal chromosomes and a delayed one that preferentially marks the sex chromosomes in late pachytene. Transgenic mice expressing only SPO11β were fully competent in establishing the first DSB wave, but late foci in sex chromosomes, which appear in concomitance with SPO11α splicing, were suppressed, leading to inefficient X–Y pairing and recombination^18^. Thus, the *Spo11* splice variants appear to operate on distinct subsets of meiotic DSBs that are both essential for gamete differentiation^18^. However, in spite of its physiological importance, the mechanism(s) underlying *Spo11* AS during meiosis are still unknown.

Herein, we describe a novel mechanism involved in the regulation of *Spo11* AS. We found that hnRNPH strongly induces SPO11α splicing. Interestingly, hnRNPH was not upregulated in pachytene spermatocytes when the switch in splicing choice occurs. However, splicing regulation was paralleled by a decrease in the RNAPII elongation rate within the *Spo11* transcription, which promoted hnRNPH recruitment and exon 2 skipping. Mechanistically, hnRNPH competed for binding and splicing of the *Spo11* pre-mRNA with Sam68, a splicing factor whose ablation causes meiotic defects^19^. Thus, our work uncovers a fine-tuned combinatorial mechanism underlying the timely regulation of *Spo11* splicing during meiosis.

## Results

### HnRNPH is a strong modulator of SPO11α splicing

**Fig. 1:**
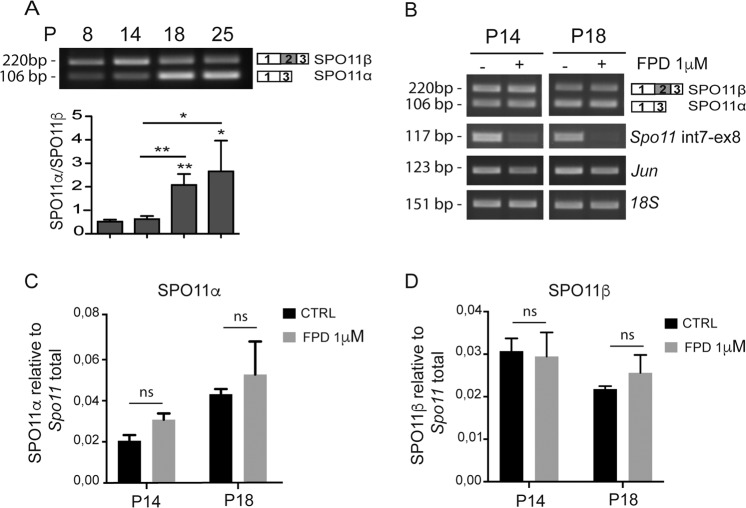
Regulation of *Spo11* splice variants expression during mouse spermatogenesis. **a** RT-PCR analysis of endogenous SPO11 mRNA in total germ cells extracted from testis at different ages (P) using primers flanking exon 2 that distinguish between the α and β variants. The bar graph represents densitometric analyses of the assay (SPO11α/SPO11β ratio; mean ± SD; *n* = 3; *P ≤ 0.05, **P ≤ 0.01, unpaired *t* test). **b** Representative RT-PCR analysis of three experiment of endogenous genes in total germ cells from P14 and P18 mice treated or not with 1 μM Flavopiridol (FPD) for 6 h. The first row represent *Spo11* alternative splicing, the second row represent a portion of the *Spo11* pre-mRNA (intron 7–exon 8 region), which is affected by transcriptional inhibition. The *Jun* gene was used as control of genes with high turnover whereas the 18S ribosomal RNA was used as normalizer. **c**, **d** Bar graph represents quantitative real-time PCR (qPCR) analysis of the level of SPO11α (**c**) and SPO11β (**d**) relative to *Spo11* total in total germ cells from P14 and P18 mice treated or not with 1 μM FPD for 6 h (mean ± SD; *n* = 3; ns not significant, unpaired *t* test).

To determine the timing of *Spo11* splicing regulation, we isolated germ cells from CD1 mice during the first spermatogenic wave (8–25 postnatal day, P), when fronts of germ cells almost synchronously enter into specific differentiation stages^20^. As observed in another strain^17^, SPO11β was the predominant variant expressed at P8 and P14 (Fig. 1a; Supplementary Fig. S1A), when testis is mostly populated by mitotic spermatogonia and early meiotic spermatocytes^20^, respectively. By contrast, SPO11α becomes the main variant at P18 and P25 (Fig. 1a; Supplementary Fig. S1A), when the most abundant cells are meiotic spermatocytes^20^. Treatment with the RNAPII inhibitor flavopiridol (FPD) to block transcription^12^ showed no significant changes in the stability of SPO11α and SPO11β mRNAs at these stages (Fig. 1b–d). These results suggest that a shift in *Spo11* splicing regulation occurs between P14 and P18.

We accurately staged male germ cells by monitoring assembly of the synaptonemal complex in chromosome spreads at P14 and P18^21^. Pachynema represents the longest stage of prophase I and early pachytene cells can be distinguished by the lack of expression of the testis-specific histone H1t, which becomes detectable in mid- and late-pachytene cells^20^. Co-staining of chromosome spreads for the synaptonemal complex protein SCP3 and H1t did not reveal major changes in the population of meiotic spermatocytes between P14 and P18 (Supplementary Fig. S1B). Thus, changes in *Spo11* AS do not reflect a clear transition in meiosis.

**Fig. 2:**
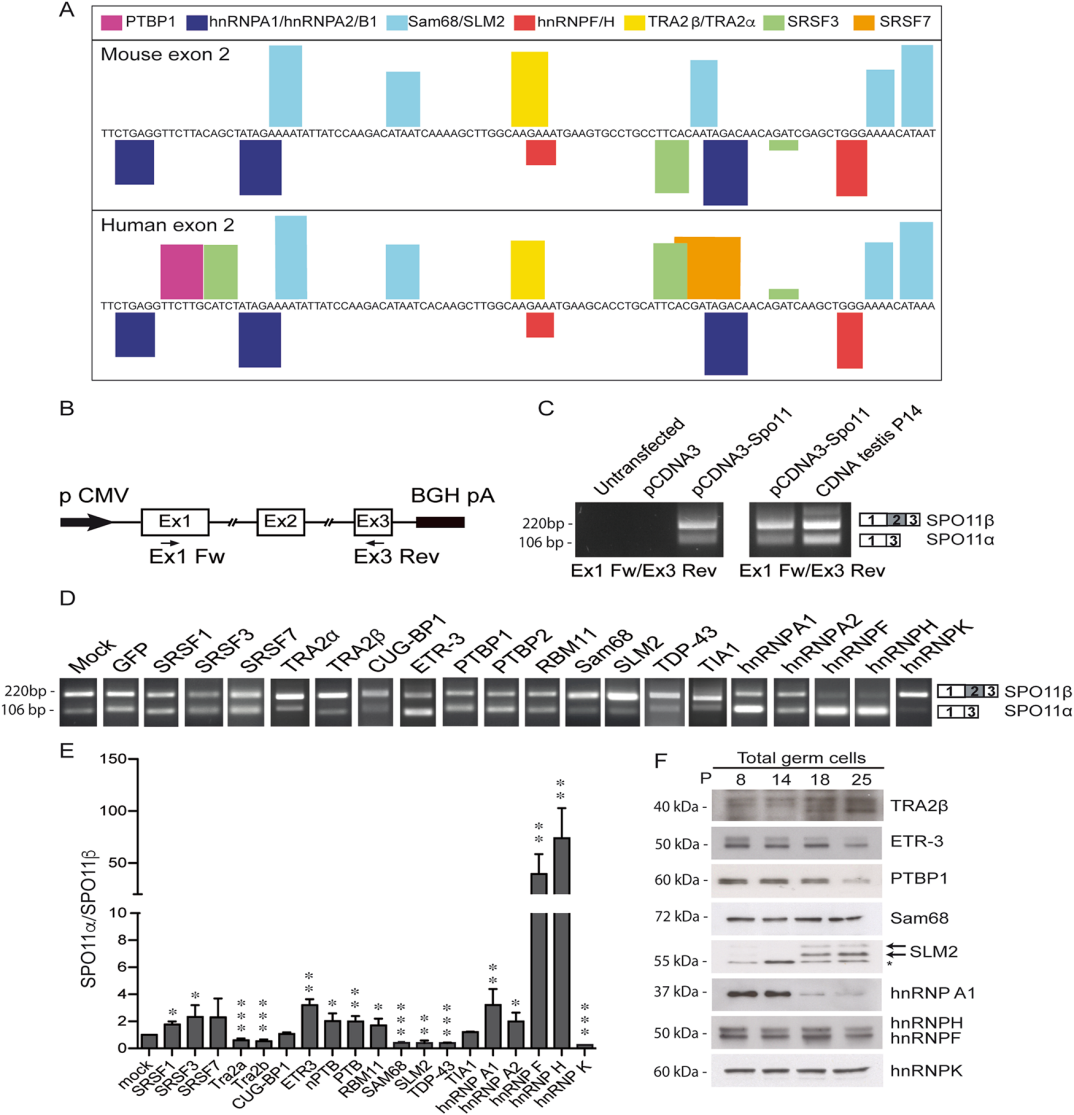
Identification of splicing factors that modulate alternative splicing of the *Spo11* gene. **a** Sequence of mouse (upper) and human (lower) exon 2 sequence showing the consensus motifs for splicing factors (SpliceAid2; http://193.206.120.249/splicing_tissue.html). Bar height represents the relative strength of the consensus. **b** Schematic representation of the minigene encoding the alternatively spliced region of the mouse *Spo11* gene from exon 1 to exon 3, including introns. **c** Representative RT-PCR analysis of five experiments of HEK293T cells, untransfected, transfected with the *Spo11* minigene or empty vector (pCDNA3) (left panel). The right panel shows the comparison with splicing of the endogenous *Spo11* gene in P14 testis. **d**, **e** RT-PCR analysis of splicing assay performed in HEK293T cells transfected with the *Spo11* minigene and expression vectors for the indicated splicing factors (**d**); bar graph represents densitometric analysis of the assay (**e**; SPO11α/SPO11β ratio; mean ± SD; *n* ≥ 3; *P ≤ 0.05, **P ≤ 0.01, ***P ≤ 0.001 related to mock, unpaired *t* test). **f** Western blot analysis of splicing factors in isolated germ cells at different ages. The asterisk indicates a nonspecific band. Coomassie blue staining of the gel was performed as loading control (see Supplementary Fig. 2C). The blots shown are representative of three images captured.

Next, we searched for factors that could promote SPO11α splicing. Analysis of binding sites within exon 2 and flanking intronic regions (100 base pair, bp) using the SpliceAid2 tool (http://193.206.120.249/splicing_tissue.html) identified several splicing factors potentially involved in *Spo11* AS in both human and mouse (Fig. 2a; Supplementary Fig. S2A). To test their effect on *Spo11* splicing, we constructed a minigene encompassing the genomic region from exon 1 to exon 3 (Fig. 2b). Splicing assays in human HEK293T cells, which do not express *SPO11*, indicated that the minigene yields both *Spo11* variants with a ratio comparable to that of P14 testis (Fig. 2c). Analysis of the predicted factors, as well as of other members of the SR and hnRNP families and of some tissue-specific splicing factors, revealed that hnRNPF and H were the strongest inducers of SPO11α splicing (Fig. 2d, e; Supplementary Fig. S2B), whereas SRSF1, SRSF3, ETR-3, hnRNPA1/A2, RBM11, PTBP1/2 elicited a much milder effect. On the other hand, TRA2α/β, Sam68, SLM2, and hnRNPK enhanced splicing of SPO11β (Fig. 2d, e; Supplementary Fig. S2B). To test if differential expression of these splicing factors could account for the switch between P14 and P18, we performed Western blot analyses on germ cell extracts (Fig. 2f; Supplementary Fig. S2C). Remarkably, none of the tested factors displayed changes in expression compatible with their effect on *Spo11* AS, as most were unchanged between P14 and P18 (i.e., Sam68, hnRNPF/H, and PTBP1) or modulated in opposite direction with respect to their effect on *Spo11* splicing (hnRNPA1, SLM2, and ETR-3). Thus, the switch in SPO11 isoforms is unlikely due to differential expression of specific splicing factors during meiosis.

### A reduced RNAPII elongation rate promotes splicing of SPO11α

**Fig. 3:**
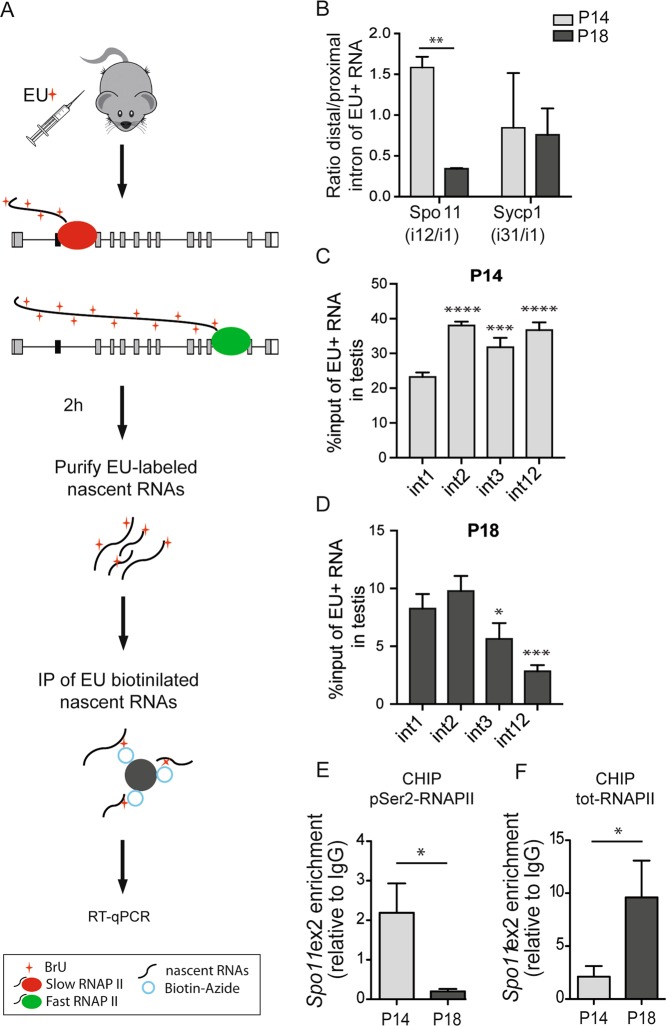
The RNAPII elongation rate within the *Spo11* gene is modulated in meiosis. **a** Workflow used for detection of EU-labeled nascent RNAs. P14 and P18 mice were treated by intraperitoneal injection of EU (300 μg/g). EU-labeled newly synthesized RNAs were collected from testes 2 h after injection, biotinilated and captured by streptavidin magnetic beads for analysis. **b** qPCR analysis of nascent *Spo11* and *Sycp1* transcripts from P14 and P18 mouse testis. The graph represents the ratio between distal and proximal intron of EU-labeled RNA. (mean ± SD; *n* = 3; ^∗∗^*P* ≤ 0.01, unpaired *t* test). **c**, **d** qPCR analysis of different regions of the nascent *Spo11* pre-mRNA corresponding in P14 (**c**) and P18 (**d**) mouse testis (mean ± SD; *n* = 3; ^∗^*P* ≤ 0.05, ^∗∗^*P* ≤ 0.01, ^∗∗∗^*P* ≤ 0.001 and ^∗∗∗∗^*P* ≤ 0.0001 related to int1, one-way ANOVA, Bonferroni’s multiple comparisons test). **e**, **f** ChIP assays of serine 2-phosphorylated (**d**) or total (**e**) RNAPII in the exon 2 region of *Spo11* in testicular germ cells at P14 and P18. The bar graph shows qPCR signals expressed as enrichment relative to IgG (mean ± SD, *n* = 3; ^*^P ≤ 0.05, unpaired *t* test).

RNAPII processivity within the transcription unit can also influence AS regulation^7–9,22^. Since RNAPII activity is modulated during male meiosis^12,23^, we asked whether *Spo11* splicing is sensitive to changes in the RNAPII elongation rate. First, we measured RNAPII processivity as the ratio between expression of a distal vs. a proximal intron^24^. To avoid issues related to intron stability in steady state transcript levels, we analyzed pulse-labeled pre-mRNAs synthesized in vivo in a limited time window (Fig. 3a). Mice were injected with 5-ethynyl uridine (EU) and its accumulation in nascent RNAs was monitored by immunofluorescence analysis. We found that a 2-h time frame was sufficient to yield efficient labeling of spermatocytes with EU in vivo (Supplementary Fig. 3A). Quantitative real-time polymerase chain reaction (qRT-PCR) analysis of EU-labeled transcripts showed that the RNAPII elongation rate within *Spo11* is strongly reduced in P18 germ cells (Fig. 3b), concomitantly with exon 2 skipping and expression of SPO11α (Fig. 1a). Moreover, analysis of other proximal regions (introns 2 and 3) in *Spo11* revealed that while all introns were efficiently represented in nascent transcripts at P14 (Fig. 3c), intron expression declined with distance from the transcriptional start site in P18 spermatocytes (Fig. 3d). These results suggest that the distal portion of the *Spo11* pre-mRNA accumulates at a slower rate at P18, in line with a reduced elongation rate of the polymerase. Such reduction was selective for the *Spo11* gene, as RNAPII processivity was unchanged within another meiotic gene (*Sycp1*) (Fig. 3b). Accordingly, by comparing P14 and P18 cell extracts we did not observe a general reduction in phosphorylation of RNAPII on Ser-2 (Supplementary Fig. 3B), which correlates with fast elongation rate^25^. However, chromatin immunoprecipitation (ChIP) experiments highlighted a specific reduction of RNAPII phosphorylation in the exon 2 region of *Spo11* in P18 testis (Fig. 3e), which likely corresponds to slower rate and pausing of the polymerase, as we observed increased occupancy of total RNAPII near exon 2 (Fig. 3f) and a decline in nascent transcript accumulation downstream of it (Fig. 3d).

**Fig. 4:**
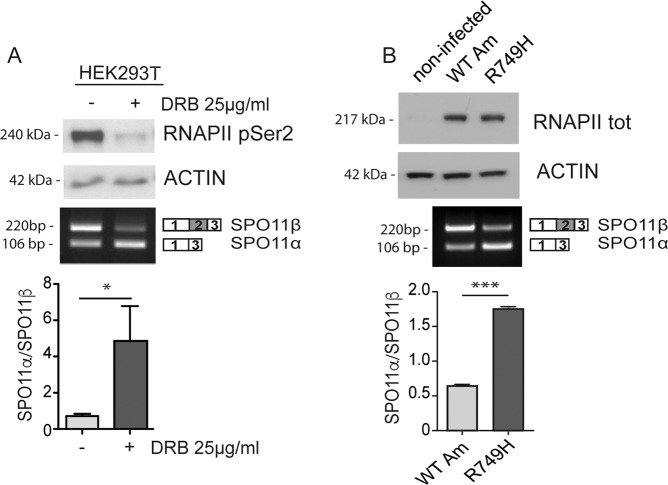
Reduction of the RNAPII elongation rate promotes SPO11α splicing. **a** Western blot and RT-PCR analyses of a representative splicing assay performed in HEK293T cells transfected with the *Spo11* minigene and treated with DRB (25 μg/ml) for 16 h. The bar graph represents densitometric analyses of the SPO11α/SPO11β ratio (mean ± SD, *n* = 3; *P ≤ 0.05, unpaired *t* test). The Western blot analysis shows the levels of serine 2-phosphorylated RNAPII in the HEK293T cells used for the assay. **b** Western blot and RT-PCR analysis of a representative *Spo11* minigene splicing assay performed in LNCaP cells stably infected with the lentiviral vector pLV-Rbp1-WT-Am^r^ or pLV-Rbp1-R749H-Am^r^ and treated with 2 μg/ml of α-amanitin for 48 h, when endogenous RNAPII was inactive (mean ± SD, *n* = 3; ^∗∗∗^*P* ≤ 0.001, unpaired *t* test). The Western blot analysis shows the similar levels of expression of recombinant wild-type and mutant RNAPII in the infected LNCaP cells used for the experiment. The noninfected cells show absence of endogenous RNAPII after treatment with α-amanitin for 48 h. The blots shown are representative of three images captured (**a**, **b**).

To test whether a slow RNAPII influences *Spo11* AS, we treated HEK293T cells with 5,6-dichloro-1-β-D-ribofuranosylbenzimidazole (DRB) to reduce Ser-2 phosphorylation (Fig. 4a) and the RNAPII elongation rate^8^. DRB treatment strongly promoted splicing of SPO11α from the minigene (Fig. 4a). To confirm that a slow polymerase is sufficient to induce this splicing switch, we engineered LNCaP cells to express an α-amanitin-resistant wild-type RPB1, encoding the large subunit of RNAPII, or the RPB1-R749H mutant, which reduces the elongation rate independently of phosphorylation^26^. The basal splicing and the response to DRB of LNCaP cells transfected with the *Spo11* minigene was similar to what observed in HEK293T cells (Supplementary Fig. 3C, D). Moreover, expression of RPB1-R749H mimicked the effect of DRB and promoted SPO11α splicing (Fig. 4b). These results suggest that changes in the RNAPII elongation rate within the *Spo11* locus modulate AS of exon 2 during meiosis.

### A slow RNAPII favors recruitment of hnRNPH and skipping of exon 2 in *Spo11* pre-mRNA

**Fig. 5:**
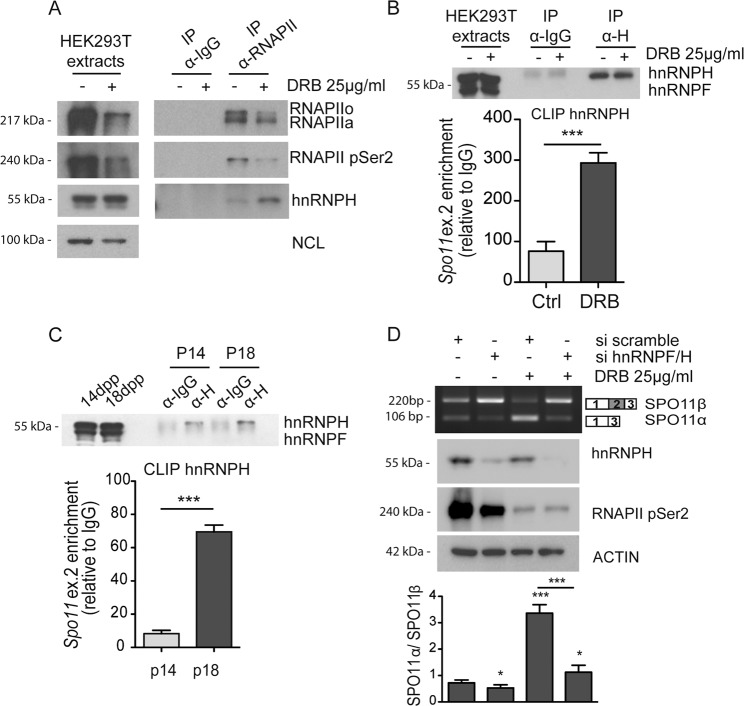
A slow RNAPII favors recruitment of hnRNPF/H in the *Spo11* pre-mRNA. **a** Co-immunoprecipitation analysis performed in HEK293T cells treated or not with DRB (25 μg/ml). Nuclear extracts were immunoprecipitated with control IgGs or anti-RNAPII antibodies in the absence of RNAse A and analyzed by Western blot for total and serine 2-phoshporylated RNAPII and hnRNPH. Nucleolin (NCL) was used as loading control for the nuclear extracts. **b** CLIP assay of hnRNPH binding to the *Spo11* minigene in transfected HEK293T cells treated or not with DRB (25 μg/ml). Cells were UV-cross-linked and immunoprecipitated with control IgGs or anti-hnRNPH antibody. The Western blot shows specific immunoprecipitation of hnRNPH. The bar graph shows qPCR signals amplified from the CLIP assays expressed as *Spo11* exon 2 enrichment in the anti-hnRNPH immunoprecipitates relative to control IgGs (mean ± SD; *n* = 3; ^***^P ≤ 0.001, unpaired *t* test). **c** CLIP assay of hnRNPH binding to the endogenous *Spo11* pre-mRNA. P14 and P18 mouse testes were UV-cross-linked and immunoprecipitated with control IgGs or anti-hnRNPH antibody. The Western blot shows specific immunoprecipitation of hnRNPH. The bar graph shows qPCR signals amplified from the CLIP assays expressed as *Spo11* exon 2 enrichment in the anti-hnRNPH immunoprecipitates relative to control IgGs (mean ± SD; *n* = 3; ***P ≤ 0.001, unpaired *t* test). **d** RT-PCR analysis of *Spo11* minigene splicing assays in HEK293T cells transfected with scramble and hnRNPF/H siRNAs. Silencing efficiency was assessed by Western blot analysis. Bar graph represents the densitometric analysis of the SPO11α/SPO11β ratio (mean ± SD; *n* = 3; *P ≤ 0.05, ***P ≤ 0.001, unpaired *t* test). The blots shown are representative of three images captured (**a**–**d**).

Phosphorylation of the carboxyl-terminal domain of RNAPII modulates its interaction with splicing factors during pre-mRNA processing^27^. As hnRNPH strongly induced SPO11α splicing, we focused on this factor. First, we evaluated whether hnRNPH interacts with RNAPII. Co-immunoprecipitation experiments revealed a weak interaction of hnRNPH with the polymerase under basal conditions (Fig. 5a). However, DRB-mediated inhibition of RNAPII Ser-2 phosphorylation promoted its association with hnRNPH (Fig. 5a), without affecting hnRNPH expression (Supplementary Fig. 4A, B). UV cross-link immunoprecipitation (CLIP) experiments demonstrated that direct binding of hnRNPH to the *Spo11* pre-mRNA was also significantly enhanced by DRB treatment (Fig. 5b), suggesting that hnRNPH association with RNAPII promotes its recruitment to the target pre-mRNA. Likewise, CLIP experiments documented that hnRNPH binding to *Spo11* pre-mRNA is increased in vivo in P18 testes (Fig. 5c), concomitantly with the reduction of RNAPII processivity (Fig. 3b) and SPO11α splicing (Fig. 1a).

Next, we asked if expression of hnRNPH is required for the effect of DRB on *Spo11* minigene splicing. Knockdown of hnRNPH, and of its close homolog hnRNPF, reduced SPO11α splicing under basal conditions. Moreover, hnRNPF/H silencing almost completely abrogated the effect of DRB on the promotion of this splice variant (Fig. 5d), indicating that hnRNPH is involved in coupling RNAPII dynamics with *Spo11* splicing regulation.

A slow RNAPII was shown to promote ETR-3 recruitment and exon skipping in the *CFTR* gene^9^. Since ETR-3 binding sites are present in the *Spo11* alternatively spliced region (Fig. 2a, Supplementary Fig. 2A) and ETR-3 weakly promotes SPO11α splicing (Fig. 2d, e), we asked whether it also acted in coupling RNAPII with *Spo11* AS. However, silencing of ETR-3 did not significantly modify the effect of DRB on exon 2 splicing and ETR-3 was bound to *Spo11* transcript in vivo only at P14 (Supplementary Fig. S4C, D). These experiments indicate a specific role for hnRNPH in the regulation of *Spo11* AS. Moreover, they suggest a combinatorial control of this event elicited by changes in RNAPII dynamics and the specific exon environment that selectively favor hnRNPH recruitment.

### hnRNPH competes with Sam68 for the regulation of *Spo11* AS

**Fig. 6:**
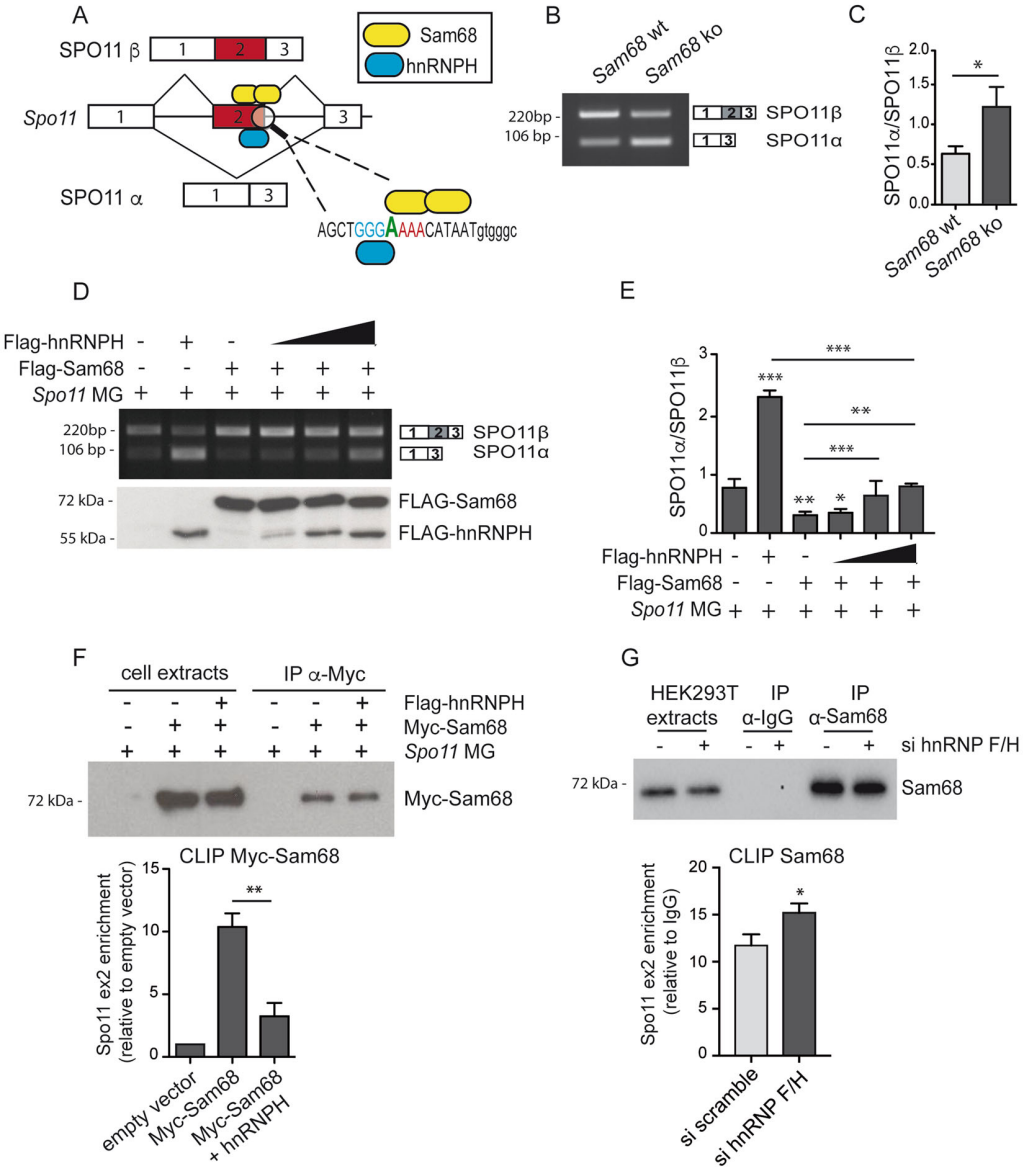
hnRNPH competes with Sam68 in the binding of exon 2 of *Spo11* pre-mRNA. **a** Schematic representation of the possible competition between hnRNPH and dimeric Sam68 for the binding to *Spo11* exon 2 near the 5′ splice site. **b** Representative RT-PCR analysis of endogenous *Spo11* mRNA in germ cells extracted from wild-type (wt) or Sam68 knockout (ko) testes. **c** Bar graph represents the densitometric analysis of the SPO11α/SPO11β ratio in germ cells extracted from wild-type (wt) or Sam68 knockout (ko) testes (mean ± SD; *n* = 4; *P ≤ 0.05, unpaired *t* test). **d** RT-PCR analysis of *Spo11* minigene splicing assay performed in HEK293T cells transfected with or without Flag-Sam68 or increasing amounts of Flag-hnRNPH. Relative expression of Sam68 and hnRNPH was detected by Western blot using the anti-FLAG antibody. **e** Bar graph represents densitometric analysis of the SPO11α/SPO11β ratio of *Spo11* minigene splicing assay performed in HEK293T cells transfected with or without Flag-Sam68 or increasing amounts of Flag-hnRNPH (mean ± SD; *n* = 3, *P ≤ 0.05, **P ≤ 0.01, ***P ≤ 0.001, unpaired *t* test). **f** CLIP assays of MYC-Sam68 binding to the *Spo11* pre-mRNA. HEK293T cells were transfected with the *Spo11* minigene and Myc-Sam68 or Flag-hnRNPH. Immunoprecipitation of Myc-Sam68 was analyzed by Western blot with anti-MYC antibody. The bar graph shows qPCR signals of *Spo11* exon 2 amplified from the CLIP assays expressed as enrichment in the anti-MYC-Sam68 immunoprecipitates in Myc-Sam68 expressing cells relative to cells expressing the MYC empty vector (mean ± SD; *n* = 3; **p ≤ 0.01, unpaired *t* test). **g** CLIP assay of endogenous Sam68 binding to the *Spo11* minigene in HEK293T cells transfected with scramble and hnRNPF/H siRNAs. Silencing efficiency was assessed by Western blot analysis (Supplementary Fig. S5). Cells were immunoprecipitated with control IgGs or anti-Sam68 antibody. The Western blot shows specific immunoprecipitation of Sam68. The bar graph shows qPCR signals amplified from the CLIP assays expressed as *Spo11* exon 2 enrichment in the anti-Sam68 immunoprecipitates relative to control IgGs (mean ± SD; *n* = 3; *P ≤ 0.05, unpaired *t* test). The blots shown are representative of three images captured (**d**, **f**, **g**).

HnRNPs often repress splicing by competing with recruitment of other factors or the spliceosome^1,4^. To test whether occupancy of regulatory sequences by hnRNPH promoted exon skipping by competing out positive regulators, we focused on Sam68 because its binding site is located in close proximity to that of hnRNPH (Fig. 6a). Sam68 promotes exon 2 inclusion (Fig. 2d, e) and it cooperates with the spliceosomal U1snRNP in exon recognition^28,22^. Analysis of testes from juvenile *Sam68* knockout mice (P10) showed a significant reduction in *Spo11* exon 2 inclusion (Fig. 6b, c), suggesting the physiological relevance of Sam68-mediated regulation. To test whether hnRNPH competes with Sam68 for *Spo11* AS regulation, we performed splicing assays with a concentration of Sam68 that induced almost complete splicing of the SPO11β variant and increasing amounts of hnRNPH. We observed a dose-dependent reversion of splicing with increasing hnRNPH levels (Fig. 6d, e). However, even at its highest dose, hnRNPH was unable to completely switch splicing toward the SPO11α variant in the presence of Sam68 overexpression.

To test whether hnRNPH competes with Sam68 for binding to exon 2, we performed CLIP assays in HEK293T cells transfected with the *Spo11* minigene. Sam68 was efficiently recruited to the *Spo11* pre-mRNA when expressed alone. However, its binding was strongly reduced when hnRNPH was overexpressed (Fig. 6f). By contrast, hnRNPF/H knockdown caused a small but significant increase in the binding of Sam68 to the *Spo11* pre-mRNA (Fig. 6g). These findings suggest that binding of hnRNPH near specific regulatory regions hinders the recruitment of Sam68, thus promoting exon 2 skipping from the *Spo11* pre-mRNA.

## Discussion

**Fig. 7:**
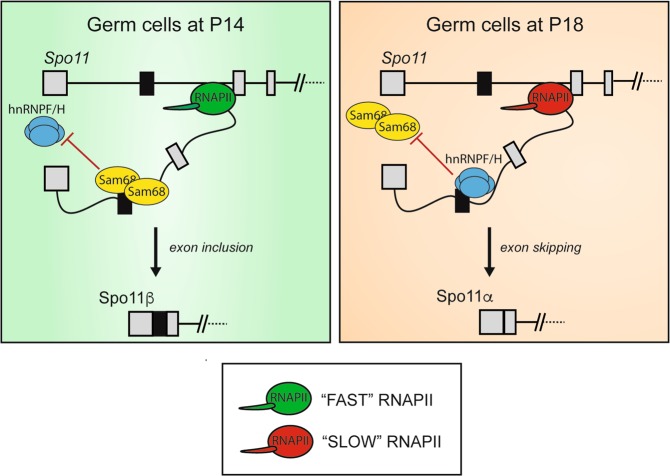
Model of *Spo11* alternative splicing regulation in meiosis. Schematic representation of the combinatorial control of *Spo11* alternative splicing elicited by RNAPII dynamics and hnRNPH or Sam68 recruitment. At P14, the fast elongation rate of RNAPII promotes recruitment of Sam68 and inclusion of exon 2, thus yielding SPO11β. At P18, reduction of the speed of RNAPII favors recruitment of hnRNPH, which competes out Sam68 and promotes exon 2 skipping and SPO11α splicing.

Male germ cells display the highest complexity in gene expression and AS regulation among mammalian tissues^10,12^. This extensive utilization of the coding potential and plasticity of the genome may underlie the unique features required for germ cell differentiation^29^. One such unique feature is the timely appearance in early meiosis of DSBs driven by SPO11^15,16^, which allow recombination, pairing and proper segregation of homologous chromosomes^30^. Notably, the main SPO11 splice variants, SPO11α and β, are differentially expressed in spermatocytes and both are necessary for proper meiosis^18^. Nevertheless, how temporal regulation of *Spo11* splicing is achieved is currently unknown. Herein, we have identified a combinatorial control that insures *Spo11* AS regulation at a specific time in meiosis, which relies on the concerted action of RNAPII and the splicing factor hnRNPH. A fast RNAPII elongation rate in early meiosis prevents binding of hnRNPH to the *Spo11* pre-mRNA, whereas the reduced speed of RNAPII in late meiosis promotes hnRNPH recruitment and skipping of exon 2 (Fig. 7). These findings uncover a novel modality of developmental regulation of splicing that couples changes in RNAPII dynamics with specific recruitment of splicing factors during meiosis, thus guaranteeing accurate control of SPO11 isoform expression in the differentiating gametes.

HnRNPH, and its close homolog hnRNPF, are potent regulators of exon 2 skipping. Interestingly, in primary germ cells hnRNPH is differentially recruited in proximity of the *Spo11* exon 2 splice site at P14 and P18, even though its expression level remains unchanged. However, we found that recruitment of hnRNPH to *Spo11* exon 2 sequences is sensitive to RNAPII dynamics, whose regulation during meiosis has been documented^12,31,32^. Several lines of evidence support this conclusion. First, interaction of hnRNPH and RNAPII is promoted by Ser-2 dephosphorylation; second, RNAPII phosphorylation in Ser-2 and RNAPII processivity within the *Spo11* locus are modulated during meiosis; third, decreased processivity correlates with recruitment of hnRNPH to exon 2 in the *Spo11* pre-mRNA; fourth, ectopic manipulation of RNAPII dynamics recapitulates regulation of SPO11α splicing in an hnRNPH-dependent fashion. These findings suggest that a fast RNAPII elongation rate prevents binding of hnRNPF/H to the *Spo11* pre-mRNA, allowing its recognition by positive regulators that promote SPO11β splicing (Fig. 7). Reduced speed of RNAPII later in meiosis, instead, favors recruitment of hnRNPH, leading to displacement of positive regulators and splicing of SPO11α. This competition-based mechanism is also supported by the observation that the strongest hnRNPH binding sites in exon 2 overlap with the consensus motif for Sam68, which promotes SPO11β splicing. Thus, occupancy of the region flanking the 5′ splice site by hnRNPH may prevent recruitment of positive regulators (i.e., Sam68) that are necessary for efficient exon 2 definition. Mechanistically, since Sam68 cooperates with U1snRNP to promote exon recognition in male germ cells^31^, binding of hnRNPH to the exon 2–intron 2 region may disrupt this interaction at P18 and cause the switch in SPO11 isoforms. Thus, our work suggests that combinatorial control of RNAPII dynamics and recruitment of specific splicing factors may dictate temporal regulation of a splicing pattern during meiosis.

The physiological relevance of most annotated splice variants remains largely unknown. In the case of SPO11, expression of SPO11β alone was not sufficient to fully compensate for the ablation of the gene^18^. SPO11β-only mice were defective in pairing of the X–Y chromosomes in late meiosis, suggesting that SPO11α plays a nonredundant role and physiologically compensates for this defect^18^. This observation also indicates that lack of proper SPO11α splicing during meiosis exposes to higher risk of sex chromosomes aneuploidy, a pathological condition that gives rise to human diseases like the Klinefelter syndrome^33^. Thus, while changes in *Spo11* expression levels can be tolerated to some extent by homeostatic mechanisms that control crossover formation^34^, lack of expression of a splice variant of the gene yields non rescuable defects. In this scenario, our findings provide mechanistic insights into the temporal control of *Spo11* splicing during meiosis and point to hnRNPH and Sam68 as key regulators of this process. Notably, *Sam68* knockout spermatocytes undergo apoptosis in late meiosis^19^. Thus, our findings also suggest that dysregulation of SPO11α expression may cause defective homologous recombination in vivo, resulting in increased meiotic cell death.

## Materials and methods

### Germ cells isolation and culture

Germ cells were obtained from testes of Swiss CD-1 mice as reported^23^. Briefly, after testis digestion with collagenase and trypsin, the cell suspension was plated for 4 h in minimum essential medium (MEM), supplemented with 1 mM dl-lactic acid, 2 mM sodium pyruvate, 10% fetal calf serum, to promote adhesion of somatic cells. Germ cells that remain in suspension were then collected. For transcription inhibition, following two washes in MEM, germ cells were released for 6 h in fresh medium added or not with 1 μM FPD (Sigma-Aldrich) and then harvested for analysis.

### *Spo11* minigene construct and PCR analyses

The *Spo11* minigene was amplified from genomic DNA of P14 mouse testis using primers reported in the Supplementary Table, cloned into pCDNA3.1(−) vector, and validated by sequencing.

For PCR analyses, RNA from samples was extracted using TRIzol reagent (Invitrogen), digested with RNase-free DNase (Roche), retrotranscribed (1 μg) using M-MLV reverse transcriptase (Promega) and used for PCR reactions (GoTaq, Promega). RT-qPCR analyses were performed using LightCycler 480 SYBR Green I Master with the LightCycler 480 System (Roche). Control reactions omitted M-MLV reverse transcriptase. Primers are listed in the Supplementary Table 1.

### Cell culture, transfections, and treatment

HEK293T cells were grown in Dulbecco’s modified Eagle’s medium (Sigma Aldrich), supplemented with 10% fetal bovine serum (FBS) (Gibco), gentamicin sulfate (50 g/ml) (Aurogene), 1% nonessential aminoacids (Euroclone), penicillin (50 U/ml)/streptomycin (50 g/ml) (Corning). LNCaP cells were grown in RPMI 1640 medium (LONZA), supplemented with 10% FBS (Gibco), penicillin (50 U/ml)/streptomycin (50 g/ml) (Corning), gentamicin sulfate (50 g/ml) (Aurogene), 1% nonessential aminoacids (Euroclone), 10 mM Hepes (Euroclone), and 1 mM sodium pyruvate (Aurogene). Transfections were performed using Lipofectamine 2000 (Invitrogen) and 2 μg of pCDNA3.1-Spo11 minigene with or without expression vectors for the indicated proteins; 6 h after transfection, cells were treated or not with 25 μg/ml DRB for 16 h before harvest. For RNA interference, cells were transfected with appropriate siRNAs (Sigma-Aldrich) using Lipofectamine RNAiMAX (Invitrogen) and harvested 48 h later for analyses.

### Pull-down assay of nascent RNAs

Testis of P14 and P18 mice were collected after 2 h of intraperitoneal injection of EU (Life Technologies) or PBS as control, as described^12^. Samples were collected in Trizol and RNA was isolated, biotinylated and captured using the Click-IT Nascent RNA Capture kit (Life Technologies). Captured nascent RNAs were retrotranscribed using the SuperScript VILO cDNA Synthesis Kit (Life Technologies) followed by qPCR analysis.

### Cross-link immunoprecipitation (CLIP) experiments

CLIP experiments were performed as described^35^. Briefly, HEK293T cells and testicular cells were irradiated on ice (400 mJ/cm^2^) in PBS, scraped off and centrifuged for 5 min at 300*g* at 4 °C. After sample processing, extracts (0.5–1 mg) were immunoprecipitated using anti-hnRNPH (provided by Prof. B. Chabot, Université de Sherbrooke, Canada), anti-MYC (Santa Cruz Biotechnology) or rabbit IgG (control) in the presence of protein G magnetic (Life Technologies). After stringent washes and Proteinase K treatment (1 h at 55 °C), RNA was isolated by standard procedures, retrotranscribed with random primers, and M-MLV reverse transcriptase (Promega) and used for qPCR. RNA associated with hnRNPH is represented as fold enrichment relative to IgG samples, RNA associated with MYC is represented as fold enrichment relative to empty vector.

### ChIP assay

ChIP was performed as described^36^ using P14 and P18 testes crosslinked in 1% (vol/vol) formaldehyde for 10 min at room temperature and quenched with 125 mM glycine for 5 min.

### Statistical analysis

Statistical tests were performed by the Student *t* test or One-Way ANOVA using GraphPad Prism6; *P* ≤ 0.05 was considered significant. No analyses were used to predetermine sample size. The experiments were not randomized and investigators were not blinded to allocation during experiments and outcome assessment.

## Supplementary information

Supplemental Information

Figure S1

Figure S2

Figure S3

Figure S4

Figure S5

Supplemental Table 1

Supplemental Table 2
