# Supplementary material for: Combinatorial control of Spo11 alternative splicing by modulation of RNA polymerase II dynamics and splicing factor recruitment during meiosis
Source: Cell Death Dis. 2020 Apr 17;11(4):240. doi: 10.1038/s41419-020-2443-y (PMC7165175; doi:10.1038/s41419-020-2443-y)
Supplement: Supplementary file 1 — Supplemental Information [file 41419_2020_2443_MOESM1_ESM.docx]

**Supplemental Information**

**Materials and Methods**

**Mouse Husbandry**

We used male Swiss CD-1 wild type and male C57BL/6 wild type and *Sam68^ko^* mice. The animals were maintained on a normal 12 hr light/dark cycle in the animal facility of the University of Rome Tor Vergata and genotyped by the Mouse Direct PCR Kit (Biotool). All applicable international, national, and/or institutional guidelines for the care and use of animals were followed. Mice breeding and housing were conducted according to the Guideline of the Italian Institute of Health (protocol n. 1088/2016-PR). We used only male mice.

**Meiotic chromosomes spreads and immunofluorescence staining**

Spreads of germ cell chromosomes were performed as described^1^. Primary antibodies used were mouse anti-SYCP3 (1:300, Santa Cruz) and guinea pig anti-H1t (1:500, a gift from Mary Ann Handel). Secondary antibodies (Molecular probes) were used at 1:200. Slides were mounted using ProLong® Gold Antifade Mounting media without DAPI (Molecular Probes). Images were captured using Leica CTR6000 Digital Inverted Microscope connected to a charge-coupled device camera and analyzed using the Leica software LAS-AF, for fluorescent microscopy.

***RBP1* lentiviral expression vector construct and lentiviral infections**

Human *RBP1* wild type cDNA was amplified from LNCaP cells and cloned into the pLenti-CMV-GFP-2A-Puro vector (abmGood.com). The R749H mutation, which slows down the RNAPII elongation rate^25^ was introduced by site-directed mutagenesis using the “megaprimer” method. All constructs were amplified using Phusion Hot Start High-Fidelity DNA polymerase (Thermo Fisher Scientific) and validated by sequencing (primers are listed in the Supplementary Table 1). For lentiviral particles production, HEK293T cells were transfected with pCMV-dR8.2 dvpr, pCMV-VSV-G and pLV-Rbp1-WT-Am^r^ or pLV-Rbp1-R749H-Am^r^ using Lipofectamine 2000 (Invitrogen). After 48 hours lentiviral particles were collected and centrifuged at 3000 rpm for 5 minutes. LNCaP cells were infected with the lentiviral vector pLV-Rbp1-WT-Am^r^ or pLV-Rbp1-R749H-Am^r^ and maintained in RPMI supplemented with 1μg/ml of Puromycin (Sigma). Infected LNCaP cells were treated with 2 μg/ml of α-amanitin (Roche) for 48 hours to inactivate the endogenous RNAPII^2^.

**Immunofluorescence Analysis of Nascent RNAs**

Nascent RNAs were analyzed by immunofluorescence using the Click-IT RNA Imaging kit (Life Technologies). P14 and P18 mice were intraperitoneally injected with 300 μg/gr of 5-Ethynyl Uridine (EU; Life Technologies) or PBS, as control, and testis collected 2 hr after injection. Samples were formalin-fixed, paraffin embedded and EU-staining performed according to manufacturer’s instructions.

**Protein extracts immunoprecipitation and western blot analysis**

Total cellular extracts from Swiss CD-1 mice testes or HEK293T cells were processed and analysed by western blot as described^3^ using the following primary antibodies (1:1000) mouse anti-hnRNPF/ H (ab10689, Abcam), anti-ETR-3 (C9367, Sigma-Aldrich), anti-FLAG (F3040 Sigma-Aldrich), anti-hnRNPA1 (R4528, Sigma-Aldrich) and anti-TUBULIN (T6199,Sigma-Aldrich), anti-PTBP1/2 (sc-56701, Santa Cruz Biotechnology), anti-TRA2β (sc−166769, Santa Cruz Biotechnology), anti-GFP (sc-9996, Santa Cruz Biotechnology), anti-MYC (sc-40, Santa Cruz Biotechnology), anti-SRSF1(sc-33652, Santa Cruz Biotechnology) and anti-SRSF3 (sc-135110, Santa Cruz Biotechnology), anti RNAPII (4H8) (sc-47701, Santa Cruz Biotechnology), or rabbit anti-SAM68 (sc-333, Santa Cruz Biotechnology) and anti-hnRNPK (sc-25373, Santa Cruz Biotechnology), anti-ACTIN (A2066, Sigma-Aldrich), anti-SLM2 (provided by David Elliott, University of Newcastle upon Tyne, UK) or rat anti-RNAPII p-Ser2 (MABE953, Sigma-Aldrich). Co-immunoprecipitation experiments were carried out using nuclear extracts from HEK293T treated or not with DRB in the absence of RNAse, as described^4^.

**References:**

1. Faieta M, Di Cecca S, de Rooij DG, Luchetti A, Murdocca M, Di Giacomo M *et al.* A surge of late-occurring meiotic double-strand breaks rescues synapsis abnormalities in spermatocytes of mice with hypomorphic expression of SPO11. *Chromosoma* 2016; **125**: 189–203.
2. Paronetto MP, Messina V, Barchi M, Geremia R, Richard S, Sette C. Sam68 marks the transcriptionally active stages of spermatogenesis and modulates alternative splicing in male germ cells. *Nucleic Acids Res* 2011; **39**: 4961–4974.
3. Fong N, Kim H, Zhou Y, Ji X, Qiu J, Saldi T *et al.* Pre-mRNA splicing is facilitated by an optimal RNA polymerase II elongation rate. *Genes Dev* 2014; **28**: 2663–2676.
4. Busà R, Paronetto MP, Farini D, Pierantozzi E, Botti F, Angelini DF *et al.* The RNA-binding protein Sam68 contributes to proliferation and survival of human prostate cancer cells. *Oncogene* 2007; **26**: 4372–4382.

**Supplementary Figure Legends**

**Figure Supplementary 1. Analysis of *Spo11* spicing in testis at different ages and male germ cells in meiotic Prophase I. Related to Figure 1.**

**(A)** RT-PCR analysis of endogenous *Spo11* pre-mRNA in testis at different ages (postnatal days, P) using primers flanking exon 2 that distinguish between the SPO11α and β isoforms. **(B)** Immunostaining of chromosome spreads for the synaptonemal complex protein SYCP3 and H1t in total germ cells from P14 and P18 testis. The pie charts represent the percentage of cells in the different meiotic stages.

**Figure Supplementary 2**. **Splicing factors analysis**. **Related to Figure 2.**

**(A)** Schematic model of exon 2 (uppercase) and portion of the flanking intron (lowercase) of *Spo11*. The upper scheme represents 100bp of intron 1 and the first 10bp of exon 2. The bottom scheme represent 100bp of intron 2 and the last 10bp of exon 2. Above and below the sequence, bar graph represented the power of binding of each splicing factors binding site. **(B)** Relative expression of the overexpression vectors used in Figure 2D was detected by Western blot using the anti-FLAG, anti-GFP, anti-PTB and anti-ETR3 and anti-MYC. **(C)** Coomassie analysis of Western Blot analysis of total germ cells at different age of development from Figure 1G.

**Figure Supplementary 3. Analysis of RNAPII elongation rate. Related to Figure 3 and 4.**

**(A)** EU staining with Alexa 594-azide of testicular paraffin-embedded cross-sections from PBS or EU-injected P14 and P18 mice. **(B)** Western blot analysis of total and serine 2-phosphorylated RNAPII in germ cells isolated from P14 and P18 mice. **(C)** RT-PCR analysis of LNCaP cells, which do not express *SPO11*, transfected or not with the *Spo11* minigene. We used pCDNA3 as empty vector for the control. **(D)** RT-PCR analysis of a representative splicing assay performed in LNCaP cells transfected with the *Spo11* MG and treated or not with different doses of DRB (10 or 20μg/ml) for 16 hours before harvest. The bar graph represents densitometric analyses of the SPO11α/SPO11β ratio (mean ± SD, n=3; ** P<0.01, unpaired t-test).

**Figure Supplementary 4. The splicing factors that can modulate Spo11 splicing. Related to Figure 5. (A)** Western blot analysis of HEK293T cells treated or not with 25μg/ml of DRB. **(B)** Bar graph represent densitometric analysis of expression level of Sam68 and hnRNPH relative to ACTIN in HEK293T cells treated or not with 25μg/ml of DRB (mean ± SD, n=3; not significant, unpaired t-test). **(C)** RT-PCR analysis of *Spo11* MG in HEK293T cells transfected with siRNA for ETR3. We verify the silencing by RT-PCR analysis of ETR3 cDNA expression and *Gadph* is used as loading control. The bar graph represents the densitometric analysis of SPO11α/SPO11β ratio (mean ± SD; n=3; One-Way ANOVA, Bonferroni's multiple comparisons test; ∗p ≤ 0.05, ∗∗p ≤ 0.01). **(D)** CLIP assay of ETR3 binding to the *Spo11* pre-mRNA. P14 and P18 mouse testis were UV-crosslinked and immunoprecipitated with control IgGs or anti-ETR3 IgGs. The bar graph shows qPCR signals amplified from the CLIP assays expressed as *Spo11* enrichment relative to IgG (mean ± SD; n=3; unpaired t-test; ∗p ≤ 0.05).

**Figure Supplementary 5. Analysis of the competition between Sam68 and hnRNPH. Related to Figure 6.**

**(A)** Western blot analysis of the HEK293T cells transfected with scramble or hnRNPH siRNAa used in CLIP assay in Figure 6G.
