# Supplementary figures and images for: Combinatorial control of Spo11 alternative splicing by modulation of RNA polymerase II dynamics and splicing factor recruitment during meiosis
Source: Cell Death Dis. 2020 Apr 17;11(4):240. doi: 10.1038/s41419-020-2443-y (PMC7165175; doi:10.1038/s41419-020-2443-y)

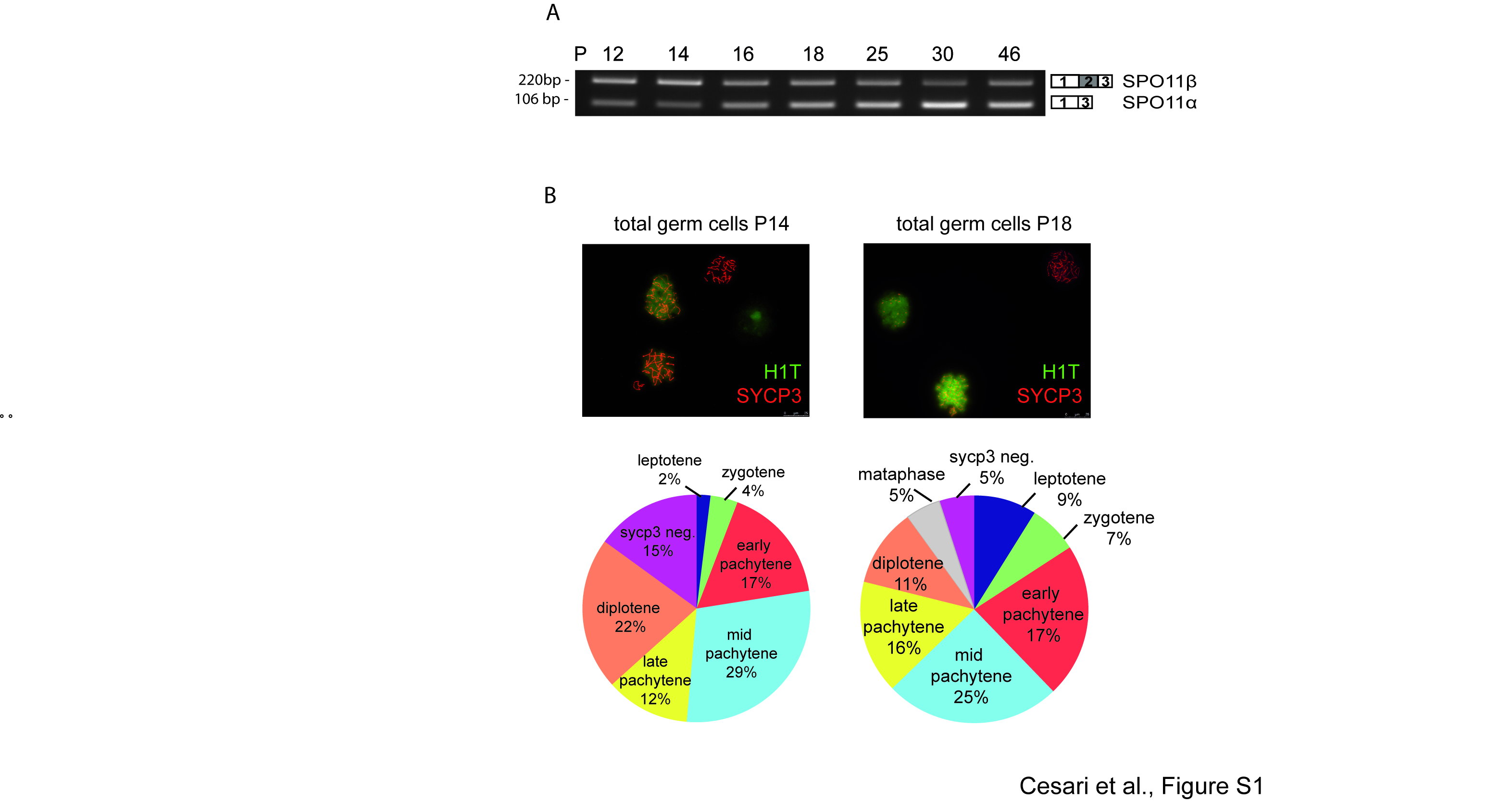

Supplement: Supplementary file 2 — Figure S1 [file 41419_2020_2443_MOESM2_ESM.tif]

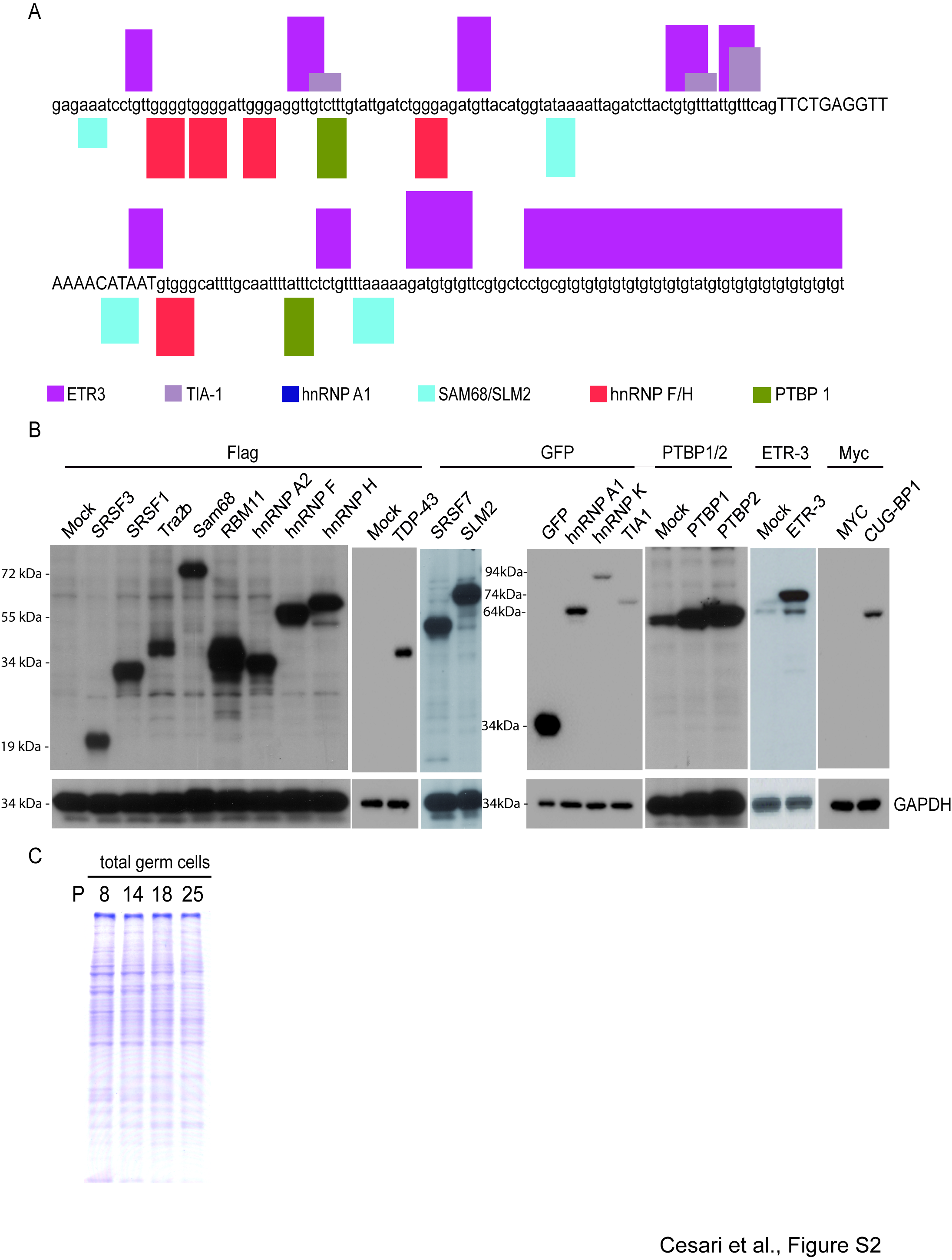

Supplement: Supplementary file 3 — Figure S2 [file 41419_2020_2443_MOESM3_ESM.tif]

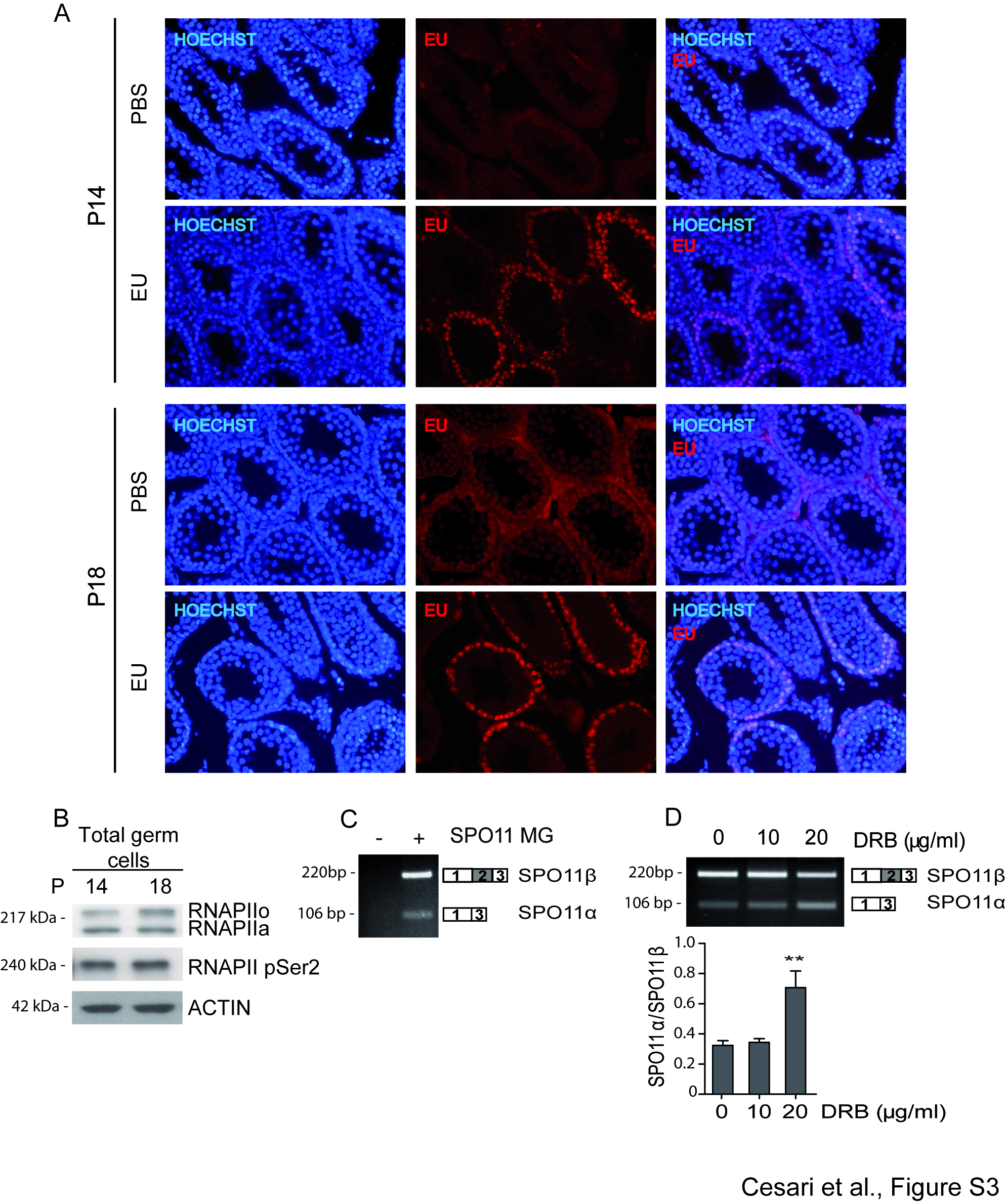

Supplement: Supplementary file 4 — Figure S3 [file 41419_2020_2443_MOESM4_ESM.tif]

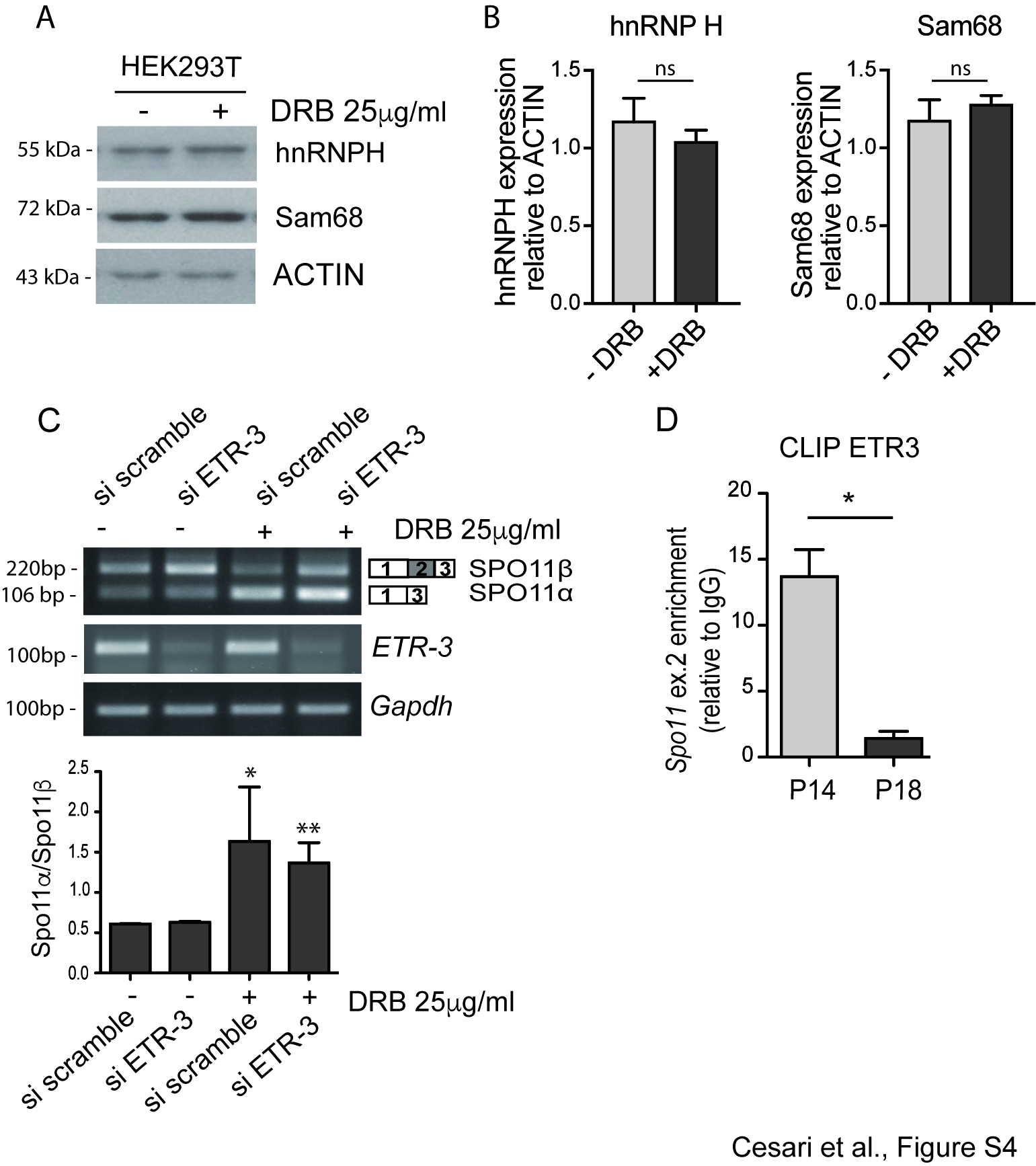

Supplement: Supplementary file 5 — Figure S4 [file 41419_2020_2443_MOESM5_ESM.tif]

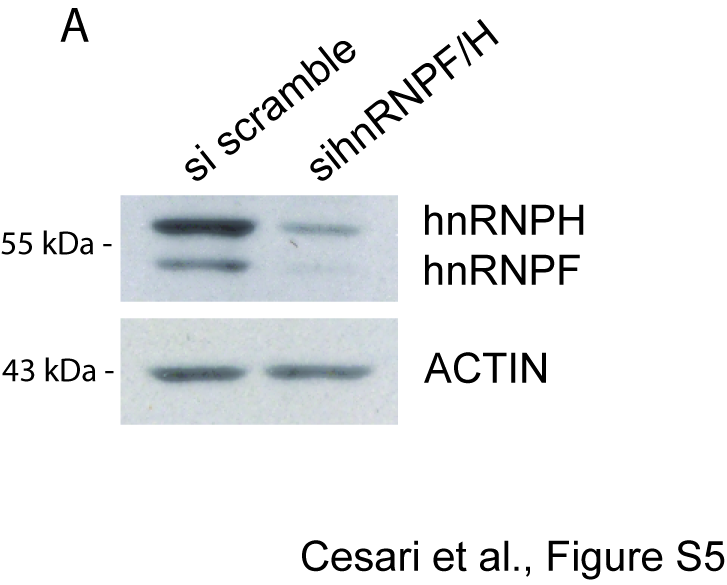

Supplement: Supplementary file 6 — Figure S5 [file 41419_2020_2443_MOESM6_ESM.tif]
